# Understanding Barriers and Facilitators of Parent/Caregiver Involvement in Home-Based Applied Behavioral Analysis Programming for Their Autistic Child

**DOI:** 10.3390/children12070850

**Published:** 2025-06-27

**Authors:** Lisa A. Ferretti, Astrid Uhl, Jessica Zawacki, Philip McCallion

**Affiliations:** 1 School of Social Work, Temple University, Philadelphia, PA 19122, USA; lisa.ferretti@temple.edu (L.A.F.);; 2 ABA Centers of America, Fort Lauderdale, FL 33319, USA; jzawacki@abacenters.com; 3 ABA Centers of America, Autism Lab, Temple University, Philadelphia, PA 19122, USA

**Keywords:** autism, ABA, parent/caregiver training, BCBA, RBT

## Abstract

There is a need for more attention to the importance of substantial parent involvement in programming for autistic children in community-based care. More encouragement is needed to ensure that practitioners prioritize parental training and involvement throughout interventions, including practitioner-led in-home applied behavioral analysis (ABA) interventions. There has been little to no research on the feasibility and efficacy of adding parental training to in-home practitioner-led ABA interventions. This study is intended to begin the consideration of efficacy by reporting on a series of focus groups involving parents of autistic children and the Board Certified Behavioral Analysts (BCBAs) and Registered Behavior Technicians (RBTs) who work with them. **Method:** Focus group meetings were conducted with a total of 18 participants: 7 family members, 5 RBTs, and 6 BCBAs drawn from two provider sites. Transcripts were generated, and data was analyzed using Braun & Clarke’s reflexive thematic analysis, a method for analyzing and interpreting qualitative data that involves systematically generating codes in order to develop themes. **Findings:** The findings are described using three main themes: (1) barriers to family involvement in applied behavioral analysis programming, (2) facilitators of family involvement in applied behavioral analysis programming, and (3) recommendations for improving family involvement in applied behavioral analysis programming. **Conclusions:** There are logistical challenges in involving parents in in-home interventions when they occur in evening hours when the family has multiple other responsibilities. However, being in-home also presents opportunities not available in school or clinic settings. The recommendations provided offer an initial road map to advancing parent training components.

## 1. Introduction

Applied behavioral analysis (ABA) is increasingly recognized as an evidence-based intervention for autistic individuals [1], and related studies have established a likelihood for increased communication, social skills, and adaptive functioning as well as decreases in challenging behaviors [2,3,4]. Such evidence has informed major growth in the number of states now mandating that health insurance coverage for autistic children includes ABA [5].

There are varying forms of ABA delivery and preferences around locations. The most common model is practitioner-led. Typically, a Board Certified Behavioral Analyst^®^ (BCBA) oversees the implementation of treatment by a registered behavior technician (RBT) who provides most of the direct intervention with the autistic child. There are also models where the parent/caregiver implements ABA procedures with their autistic child to assist with acquiring new skills and/or decrease challenging behaviors. In these models, the BCBA provides training, support, and coaching to the parent/caregiver. Several studies have documented not only improvement for the child but also health- and stress-related improvements for the parent/caregiver implementing program activities (Postorino et al., 2017 [6]; Rodgers et al., 2021 [7]). This includes findings that parent/caregiver-led ABA is as effective as practitioner-led [8,9]; however, the delivery trends are for practitioner-led delivery. Nevertheless, there has been growing attention to findings that parent/caregiver-led interventions lead to significant increases in parent/caregiver self-efficacy [10] as well as child outcomes.

Tabatabaei et al. (2022) [11] in their review of parent/caregiver-involved autism intervention studies emphasized that autism has consequences that affect all aspects of a family, with parents/caregivers experiencing more anxiety and lower quality of life than parents/caregivers of children with other disabilities and with none. They argue that involvement in interventions helps parents/caregivers cope and that the successful provision of assistance and the sense of building skills offers hope [11]. The review found evidence of positive impact from parent/caregiver involvement in interventions on the autistic child’s social relationships and behaviors, and reductions in repetitive behaviors. From the parent/caregiver’s perspective, they reported effects on increasing parent/caregiver self-confidence and their satisfaction with interventions being undertaken as well as on enhancing the parent/caregiver’s ability to implement interventions [11].

In another systematic review of all types of parent/caregiver-led interventions for autistic children, ref. [12] summarized studies that found such interventions increased parent/caregiver skills and knowledge; encouraged parent/caregiver–child engagement; addressed behavior support, communication, and social interaction needs; and increased daily living skills. Twelve well-designed studies reported success in improving social skills and other pro-social behaviors, eight addressed problem behaviors, six increased language/communication, and twenty-five studies addressed both social/positive behaviors and language/communication. Equally important findings in several studies were that instruction, role play, and coaching/supervision through in-person or virtual home visits, including by BCBAs, proved critical to parent/caregiver success (see, for example, [13,14]). In addition, parent/caregiver training was found to compliment professionally led programs by being a mechanism to generalize to daily routines and expand the frequency of delivery of proven interventions provided (see Bearss et al., 2015 [15]; Dawson-Squibb et al., 2020 [16]).

The combination of all these findings has been interpreted as encouraging more attention to the importance of substantial parent/caregiver involvement in programming for autistic children in community-based care and encouragement that practitioners prioritize parent/caregiver training and involvement throughout interventions, including in practitioner-led ABA interventions [10] (Sneed & Samelson, 2022).

Turning to location, ABA sessions usually occur in clinics or classrooms but increasingly are being mainstreamed into the autistic child’s own home. Among the reasons for these transitions are that ABA provides real-time opportunities to address stressful situations where they occur, particularly in the home; enhances delivery by occurring in a familiar place; presents an opportunity for undivided attention from the practitioner, particularly the RBT; builds afterschool homework and playtime independence; supports a focus on in-home behaviors such as meal-time and bedtime routines; and potentially builds relationships with parents/caregivers, siblings, and other family members in ways that support ABA plans, goals, and activities. Yet there are challenges as in-home sessions usually occur after school and in the evening when the family is often at its busiest with the return of parents/caregivers perhaps from work, mealtime prep, and support needs of other siblings in the home. On the other hand, in-home services offer an expanded opportunity for direct and personal training for parents/caregivers.

Good practices are increasingly encouraging parent/caregiver training, and insurance is supporting such extensions of what is offered. However, as Sneed and Samelson (2022) [10] note, there has been little to no research on the feasibility and efficacy of such hybrid approaches of practitioner-led ABA interventions with added parent/caregiver training. This study is intended to begin the consideration of efficacy by reporting on a series of focus groups involving parents/caregivers of autistic children and the BCBAs and RBTs who work with them. It will also address barriers to participation, facilitators of participation, and recommendations for improving parent and caregiver participation in applied behavioral analysis programming.

## 2. Materials and Methods

Focus group meetings were conducted by two of the authors (PMC and LAF) with volunteer participants. Steps were taken to ensure that (1) all individuals consented to their participation, (2) all reporting was anonymous, and (3) an atmosphere was created where participants felt they could speak freely, with the understanding that there would not and could not be any negative consequences for their receipt of services. Sessions lasted approximately 60 min and were recorded with participants’ consent.

The original plan was that interviews would be conducted in person, and a number of participants did indeed participate as in-person one session focus group members, but others chose to complete the session as a group via a secure Zoom link. This reflected the busy lives of the involved parents/caregivers in particular. Once there was a sense (agreed by both researchers) that saturation of perspectives was achieved, the groups stopped. There was a total of 18 participants: 7 parents, 5 RBTs, and 6 BCBAs. Of the participants, 75% were white, 25% were Black or Latinx, 28% were male, 72% were female, and all were aged between 20 and 50 years.

*Parents* interviewed had school-aged autistic children, and many had struggled to get confirmed diagnoses for their children (several raised and expressed their appreciation that ultimately it was through the ABA provider that the necessary assessments were completed and they were able to obtain a diagnosis). They were a mix of families, three of whom had received ABA services for approximately three months and four for whom services had been provided for over one year. All were receiving at least four days of services each week.

*RBTs* had been delivering ABA services for at least a year, several had experience from working at more than one ABA provider, and most had a small case load.

*BCBAs* had multiple years of experience, including experience at more than one provider, and were supervising multiple RBTs.

Community providers and parents were consulted in the development of the research methods, and a similar semi-structured interview guide was utilized during the focus group discussions with each sample.

Analysis Approach: A transcript was generated from each focus group session, and the transcripts were then the basis for analyses. Two researchers (LAF and AU) reviewed the transcripts for accuracy, and where necessary, audio files were re-listened to in order to ensure that what was surmised from the transcripts genuinely reflected what participants were seeking to convey.

To generate coding categories and subcategories in line with the aims of the study, interview data was analyzed using Braun & Clarke’s reflexive thematic analysis [17,18] (Braun & Clarke, 2006, 2022), which is a method for analyzing and interpreting qualitative data that involves systematically generating codes in order to develop themes. Members of the research team read and re-read the interview transcripts, building familiarity with the materials, making notes/memos, and generating initial ideas from the data.

Once the interview transcripts had been re-read and notes/memos discussed, the researchers began to generate codes with relevance to parent/caregiver training. An inductive approach was utilized in which the research team members generated initial codes based on the responses using a bottom-up approach to analysis. During the analysis process, four-step qualitative data analysis strategies [19] were used. The first step involved identifying codes or “meaning units” from the participants’ answers to the questions in the interview form. Codes generated were then clustered with similar ones into a category or theme, while dissimilar codes were separated to create distinct categories. In the second step, codes were sorted and placed in emergent categories, and the categories were then examined for themes or patterns. In step three, the categories were further examined for meaning, interpretation, and potential for support among the groups interviewed. In step four, a diagram was constructed to illustrate the codes and themes found in the data [20].

**Figure 1:**
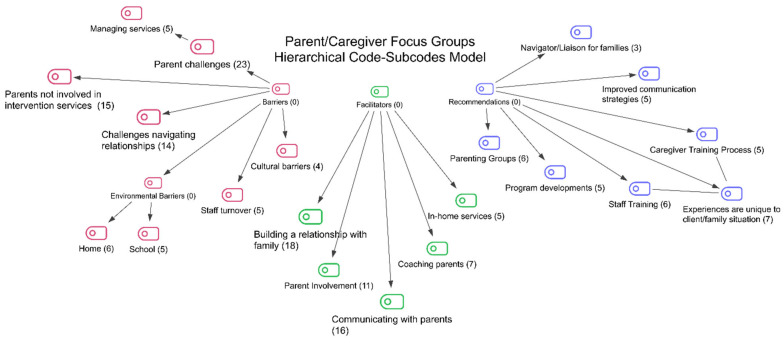
Note: Number in brackets after subcode name is number of mentions of this subcode.

Codes were developed and modified throughout the coding process using the qualitative data analysis software MAXQDA 24 (MAXQDA 2024 VERBI Software, 2024) [21]. Once all the data had been coded, the team members began to iteratively generate themes that described patterns observed in the data that could be used to further group similar codes together and to look for opportunities to meaningfully create overall themes that cross-cut the three groups interviewed. Code and themes lists were generated, and after several iterations, the codes and initial themes fell under three main themes: (1) barriers to parent/caregiver involvement in applied behavioral analysis programming, (2) facilitators of parent/caregiver involvement in applied behavioral analysis programming, and (3) recommendations for improving parent/caregiver involvement in applied behavioral analysis programming. These themes and related codes are shown in Figure 1.

## 3. Results

The results are described using the three major themes that emerged.

### 3.1. Barriers to Parent/Caregiver Involvement in Applied Behavioral Analysis Programming

As noted in Figure 1, barriers identified included challenges in navigating relationships, cultural barriers, environmental barriers in the home and at school, parent/caregiver challenges, the lack of involvement of parents/caregivers in intervention services, and staff turnover (see Table 1 for definitions). Parents/caregivers were most likely to identify their own parent/caregiver challenges (the challenges they face as parents/caregivers of an autistic child and in managing the disconnect between home and school), but some did raise the issues of busy schedules, their own or a partner’s lack of engagement with the ABA services and, in several cases, concerns about staff turnover. Most barriers were identified by parents/caregivers and by the RBTs.

One BCBA summed up the challenges in relationships as follows:

I mean, I genuinely think it’s the hardest job in the whole world. Like, I’m not exaggerating. I think working in people’s homes is so hard in so many ways. And you’re always going to have, you know, different personalities and families that do things differently than what you think they should be doing things. So we’re constantly having to rewire our brains and come up with creative ways of, like how to incorporate programming or communicate effectively. I think that it’s the hardest part of our job.

An RBT agreed with their peers by saying

Because this is not our child. I, you know, I’m not a parent/caregiver, so I have much less, you know, real world experience. And sometimes there can be friction between what the family’s wishes are and what is clinically appropriate or even just possible. Right? There’s some families that are incredibly accommodating, and they understand this, the severity and the impact that their child might have, and then other ones are more or less looking for almost like a miracle where they, they want this to just go away.

Finally a parent/caregiver received nodding affirmation from other parents/caregivers when they said

Sometimes it feels a little invasive. It’s, you know, not that they’re a complete strange After a period of time, but you know it’s like having a friend over at your house. You may enjoy their company, but at the same time you’re like I want my space in my house, and the duration of time could take a toll on you, and you’re sitting there worried about oh, no, we gotta get the dishes done. How do I present my house in a time and in manner that’s like respectable, and I don’t wanna be judged. Not that anybody’s judging me on how my house looks at the moment, but it can wear on myself and my wife and it will take a tol.

All appeared to agree that there is work to be done on building relationships before the parent/caregiver training component can be approached. An area of concern in the early days of building relationships is negotiating cultural issues. It was interesting that only BCBAs raised this issue. As one BCBA noted and others affirmed,

What I’ve noticed mostly is one I don’t have children, so parent/caregivers find it difficult to think that I can’t relate to them because I don’t have children yet. And another difficulty I’ve had is also like a language and kind of cultural barrier. I’m from (a country other than the United States) so a lot of the things that happen here in the US, I’m still getting used to, so there’s that cultural barrier. And then I have had. English isn’t their first language, and that communication barrier is there, so it’s just finding a ways around that.

Environmental barriers were mentioned by both BCBAs and RBTs. An RBT noted

Challenges with each location. In the home, typically, this is a very familiar place for the client, and it can be difficult to get them to attend when their bed is right here. When you know brother and sister are watching TV. And you know the parent/caregivers are doing their daily activities. Not every family is in the position to have multiple rooms, so it can be a little cramped to actually get some time secluded away from all the action

Parents/caregivers were more likely to talk about challenges with the school, which meant a loss of gains at home.

He seems to improve at home. We still have a lot of issues in the classroom so he doesn’t seem to be transferring the skills that he’s learning into his general education classroom.

A BCBA sympathized with the parents/caregivers’ dilemma and concerns.

I think a lot of times parent/caregivers don’t know the right questions to ask and they get a lot of information from the school, and it’s either not explained or it’s incomplete. And then they see us working at home. And you know we work on different things sometimes in school and home, and I think there’s a big disconnect.

Parents/caregivers spoke of the challenges in having someone coming into their home:

It’s really a time you feel uncomfortable. Somebody’s in your space that you’re not used to being in your space. Not only that, but you don’t know them. So it’s almost like a new relationship where you’re like: I want to love you. I want to. I want to make this work. But it’s, it’s tricky. I would say. It took a good solid couple of months before I finally felt like I could act like myself. and ha! Feel completely comfortable with having them in our home.

Managing services is also a challenge:

…We’re all kind of pulling our hair out. So I feel like they’re doing as much as they can right now. And they’re actually- our BCBA is having a meeting with our school BCBA next week to kind of try to get on the same page in terms of the plan for the potty…

For BCBAs and RBTs the parents/caregivers not being involved in interventions was a particular challenge. A BCBA noted

…some caregivers get attached to, you know, staff, like what we do, that they believe that they can’t do it themselves. So they feel like, you know, if we are here, we’re the ones that are helping the client, but it’s like they don’t want to believe that they can also do it themselves. That’s why I also like the modeling piece because it’s like, hey, you know, I know we talked about this all the time, but I want to see you implement. I want to see you do it But I know some caregivers too, they have a hard time like- It’s like we always like to say, like, you know, we don’t want to be here forever. We just want to help them to a certain point where they will be good to go. But I think caregivers, when they hear that, they’re like, no, I don’t want you guys to go…

A parent/caregiver noted

So how will you know? Let me know how his sessions with his ABA visitor goes; usually I’m working remote in grading or having meetings with students while he has his sessions in his room

An RBT summed up the challenge:

The reality is that most family members work a little bit during session, right? If session goes from 4 to 7. Maybe Dad gets home at 5, and then he can participate for an hour or 2. But you know, when there’s vacation days and daytime hours. It’s just not realistic to expect them to always be there.

Parents/caregivers also raised the challenge of staff turnover:

So we reached a point I was like, listen, we need like 2 consistent RBTs because this isn’t working for him. And then we had that conversation, and and they made it happen so.

A BCBA explained why staff turnover is so problematic:

So I think when our training is different or our approaches are different, it poses an extra barrier for families because they get accustomed to the way things were done, and then it changes

### 3.2. Facilitators of Parent/Caregiver Involvement in Applied Behavioral Analysis Programming

As noted in Table 2, a range of facilitators of parent/caregiver training were identified and are defined in Table 2: building a relationship with the family, coaching parents/caregivers, communicating with parents/caregivers, the opportunities offered by in-home services, and parents/caregivers becoming involved. It was noticeable that BCBAs were most likely to offer ideas on facilitators.

A BCBA described the situations where parent/caregiver training is likely to be successful:

I feel like one constant thing that we constantly remind the RBTs of is pairing, pairing with the clients, pairing with the parent/caregivers when they’re in the house. They don’t have to do that as much in the clinic setting, but I feel like it’s very important to pair with the family and the clients in the home and understanding their values and what’s important during the sessions. Cause the RBTs, they wanna go in and they think that doing a good job means like, I need to look at this iPad and I need to check off all the boxes. But really they need to also foster that relationship and understand what’s important to the families

A parent/caregiver discussed how the relationship building benefits their autistic child:

He’s accepted what we’re doing. Now, it’s more play based. But I’m okay with it because he is opening up. He trusts them? They’ve, they’ve built- They built a relationship with him at this point. So now we’re working on some of those things,

There was also appreciation by parents/caregivers of the coaching approaches used:

But the BCBA in particular, really, for me, for my experience has really pointed things out that were really eye opening as a parent/caregiver. Like she one time was just in the kitchen we were talking, and, you know, cause things unfold, you know, at random in this experience. But she pointed out to me, she’s like, do you know how many questions you just asked him? And she’s like, do you know how many words you just used? And I was like, Oh, my goodness! So like it’s things like that that. I didn’t realize And I’m like Whoa, like I was mind blown like I know I like that is just counterintuitive as a parent/caregiver like I’ve never know how to do things that way. So they are definitely coaching

Another parent/caregiver talked of:

The constant back and forth with the BCBA of like, you know, we’re working on this. Okay, now, what else concerns you? Let’s jot it down. Let’s make a game plan of what else we can do better so and then and then implement, and then just being around them and implementing the different things that I see them do like, I will try and use them when they’re not home or when they’re not in my home.

A BCBA emphasized the importance of such moments:

And then there’s another, you know, form of parent/caregiver training. That is that in the moment, in vivo with their kid, kind of like, okay, we talked about this in parent/caregiver training. Now, this is how we’re gonna- this is how we’re doing it. And it can make it naturalistic with the child and the parent/caregiver like, hey, why don’t you try that like this functional communication. Why don’t you try this play? You know, taking turns and turn taking and incorporating that that in, where it again, it looks more naturalistic.

Success and facilitation did seem to come down to communication, particularly for BCBAs. A BCBA spoke of:

A lot of what we wanna do is naturalistic teaching, and we don’t wanna make it look like work. So the parent/caregivers aren’t trained like we are. So it’s really important to show them as it’s happening like the little nuances that we’re sprinkling into a session

Another BCBA argued:

Conversation is just the key component in what we do. We always have to reiterate, even if it’s just the first week we’re starting with the client or you know the client for two years, always have to reiterate like you know, our terminal goal, the caregivers goals, like at the beginning we should be asking the caregivers what are your goals for your child? do you want to see them communicate more? You want to see them engaging with other peers? So over time we are going to be taking data on those things. And that’s why we love data. That’s why we like to show like, hey, this is, you know, what we’re working on, it’s working.

BCBAs also spoke of the opportunities from working in home settings:

Being home based now, which is also new to me, it I’ve noticed that it gives the parent/caregivers the support that they’ve never really experienced before, especially for parent/caregivers with the younger children. They’re just getting the diagnosis. They don’t know what to do. Having someone else, there are RBTs and myself in their home. They have someone who can, they can communicate with and just, you know, experience all the different joys and pitfalls of just raising their child and helping their child gain skills that you know, other children have an easier time gaining skills with.

An RBT described the unique joy and opportunity in involving parents/caregivers and other family members:

If they can see me acting as a model for the way to appropriately deliver these. I’m over the moon when you know grandma wants to try to get in there and do the same thing because they’re gonna get so much more contact with their, you know, relative. I’m just some guy that comes to their house. Their grandma is going to be someone they look up to and wanna respect their wishes a lot more.

A BCBA reinforced these feelings:

When we’re in the home, they get to observe all that, you know, they get to observe how we do toilet programs. They get to observe how we do meal time. They get to observe, observe how we do. We even play like the kid like, you know? So it’s like sometimes they Say, ‘wow, I didn’t think of it. I didn’t think to play with them like that’, you know? And some like I say, sometimes it can be the simplest thing, but it’s like they need to see it. And that’s why I love modeling. I love being there

### 3.3. Recommendations for Improving Parent/Caregiver Involvement in Applied Behavioral Analysis Programming

As noted in Table 3, parents/caregivers, RBTs, and BCBAs offered a number of recommendations for caregiver training, valuing the uniqueness of individual situations, improving communication, developing a navigator/liaison role, parent/caregiving groups and additional programming for families, and offering more training for staff. All three groups were well represented in offering recommendations.

RBTs and BCBAs had a number of recommendations around caregiver training. There were the suggestions of fact sheets and flow charts.

An RBT noted

One thing one of my BCBAs does, and they’re amazing at it, is they’ll write out this template that goes through the different phases of escalation with a client, but like some of the cues that are occurring and what exactly to do in those instances for both the RBT’s and the parent/caregivers.

A BCBA stated

I think caregivers respond to different styles, too. So I feel like that kind of goes back to pairing like you figure out what they’re going to respond best to.

One parent/caregiver stated

I think for me the most possible thing would be to understand the programming like, what are they gonna be doing with my child every day.

BCBAs collectively emphasized the need for understanding that experiences are unique to client/family situations. One spoke for many with this example:

I’ll also say that really depends on the client. It really depends on the programmers that you have in place. We have certain clients that receive services after school, so they’ve been in school all day and you’re coming in their home at 4:00. So you may not the minute you get there, you may not jump right into session. You may give them a break to, you know, decompress. You may be engaging in like deep conversations. How was your day? What did you do today? And then do like one or two programs. Like it really just depends on the client because everybody’s different

Improving communication strategies coupled with a family concern at times that they had to go through the BCBA to talk about the programming for their child when the RBT was right there in their home and their questions were about what the RBT was delivering.

One parent/caregiver expressed

The RBTs are the ones that are in our homes 90% like they’re the ones that are here with us 100% of the time during session. And there have been so many times where my little guy is having like a brutal day, or something happened that I absolutely will not tolerate bringing up again in front of him, for whatever reason it may be. And don’t have the option to directly speak to my RBTs, give them a heads up, ask a question. I have no way to do that if I need to say something to my RBT, I have to go through the person that does our scheduling, which I find is absolutely ridiculous. I don’t like that at all. I don’t like the fact that I can’t just drop a message and not have to include a third person

Other parents/caregivers agreed. As one said

We can text with our BCBA and we talk a lot with our scheduler. But not being able to talk to the RBT is hard. It’d be nice to like, just feel like, get texting. Give a heads up about like what’s going on today, just setting kind of the tone for what’s happening? I don’t know. Yeah, I hadn’t thought about that a lot but now that it’s like brought up, Yeah, it would make things easier for sure.

Parents/caregivers also recommended that there be consideration of the development of a navigator/liaison role:

It would be nice to know if there was some sort of maybe not support right word. But someone that kind of I don’t know like explains not even necessarily the science behind the program, but just like what this does and why this is important.

Another parent/caregiver said

Almost like a counselor in a way to help you navigate some certain like certain situations, because it’s not a one size fits all and a lot of families do get- they’re like, okay, this, I mean, this doesn’t make sense. This doesn’t make sense there’s always a lot of questions always arise and it would be nice to have an opinion or somebody- an ear, somebody to talk to that can put your mind at ease and help navigate if needed.

Parents/caregivers also raised their need/desire for parent/caregiving groups:

I think, like a parent/caregiving peer group, you know, with everyone that is receiving services. I think it’d be beneficial to the parent/caregivers to be like. ‘Oh, other people out there have a child like me. How do they support their child at home?’ And it would be- they’d be able to express their feelings and their struggles, and maybe someone has found a way to help their child and can give ideas to the other family on possible ways to help them succeed with their child.

A different parent/caregiver said

I’m like, just it’s been constant learning, like it is just endless. So I mean, I’m all for like the more I can learn, the more I can share. You know, even if it was on like a monthly basis, or I don’t know how often, but I think it would be something beneficial, because I think we all- I mean I don’t know about anybody else- But there is a financial aspect of this that was incredibly scary, and I could share kind of my journey, if that helps someone else, I think it’d be like worth it.

Finally, parents/caregivers spoke of their need for additional programming:

One thing that we have a really hard time with- so he he’s not qualifying for extended school year and finding camps who are willing to take on a kid who is autistic is really hard. Even if they’re in like a general ed classroom like they see that diagnosis like no. So maybe like a summer camp or something or a week of like full time camp would be great, so they can still get the benefit of that socialization.

BCBAs and RBTS also had additional recommendations in the area of staff training that they felt would help further build rapport with families and support parent/caregiver training.

A BCBA thought

I think, like cultural sensitivity training or cultural understanding training would be would be nice. Because we do get, you know, we have a lot of families with different backgrounds and different you know beliefs. And I think that I think that that would be helpful.

An RBT shared

More speakers that are actually like adults with autism, living that life, who have found success in their own way and kind of bring in that experience And we get to experience that as the other side of okay, we are the people who support, you know, autistic people. How can we do that better, learning from the experience of someone who has autism.

## 4. Discussion and Conclusions

Parents/caregivers tended to understand the role of applied behavioral analysis interventions for their autistic child and welcomed and appreciated the important change ABA made in their lives and in their child’s life, but some had difficulties with adjusting to the intrusion in their lives of an intervention that occurs during prime family time and for multiple evenings. Parents/caregivers also expressed a lack of clarity on what their role was and difficulty with “rules” around communicating with RBTs as opposed to BCBAs. Consistent with the Tabatabaei et al. (2022) [11] review findings, the families reported that the interventions helped them cope but there were no reports of them building skills in the same way as their findings suggested.

RBTs were clear about their roles and the need to work with family members, and they appreciated the support they received from BCBAs in negotiating with parents/families and in addressing family dynamics that they do not see as helpful to their interventions. BCBAs believed that they were delivering parent/caregiver training in their interactions with parents, but parents do not always see BCBA input as parent/caregiver training nor did they fully understand why parent/caregiver training was part of the intervention they received for their child. The differing views and lack of clarity made it more difficult to understand the barriers and facilitators to parent/caregiver training in practitioner-led, in-home ABA interventions.

Nevertheless, barriers were identified, most frequently by parents/caregivers and RBTs, and included challenges in navigating relationships, cultural barriers, and environmental barriers both in the home and at school; parent/caregiver challenges; the lack of involvement of parents/caregivers in intervention services; and staff turnover. The wording of the barriers again highlighted the misunderstandings of roles, as well as the unique issues that arise when there are cultural differences, when in-home delivery produces discomfort for parents/caregivers and RBTs and when services occur at a time of day where families have other commitments and welcome RBT activities as a respite rather than as a joint enterprise. All acknowledged the types of benefits of ABA training, including improvements in communication, social skills, and adaptive functioning that have been described in the literature (Irwin & Axe, 2019 [2], Makrygianni et al., 2018 [3]; Wong et al., 2015 [4]).

Communication rules were also seen by parents/caregivers as disrupting the potential for bonding and collaboration. It was noteworthy that BCBAs in particular often used the same pairing language as they used to describe a critical pre-requisite to delivering ABA with autistic children when also describing building relationships with families but did not seem to have a solution for the parent/caregiver view that they were being prevented from pairing with the RBT in their home. Changes in practices and/or in language may be needed to address this particular barrier. More understanding of the challenges for parents/caregivers in evening hours (when interventions are often for 4-5 evenings per week) may also be required.

Yet there were several facilitators also identified: building a relationship with the family, coaching parents/caregivers, communicating with parents/caregivers, the opportunities offered by in-home services, and parents/caregivers becoming involved. These tended to be described by BCBAs only and perhaps reflect the disconnect or poor communication between BCBAs, RBTs, and parents/caregivers and their different viewpoints and priorities rather than an unwillingness to work together. Here too, greater communication and clarification of roles may be helpful.

The reality that parents are overwhelmed by autism, managing both school and home for their autistic child, and managing work and home for themselves and other children was reflected in their recommendations for developing a navigator/liaison role, parent/caregiving groups, and additional programming for families. Equally, BCBAs and RBTs were thoughtful in their increased likelihood to recommend caregiver training, valuing the uniqueness of individual situations, improving communication, and offering more training for staff. All of the recommendations seem feasible, although which mechanism would pay for what intervention would need to be negotiated.

### Limitations

There were some limitations in this study in that all families received their ABA interventions from the same agency, so generalizability cannot be assumed. Also, the majority of parents/caregivers interviewed had been receiving ABA services for less than two years, meaning that the same findings cannot be assumed for more experienced families.

The promises previously ascertained for parent/caregiver-led or involved interventions of improvement for the autistic child and of health- and stress-related improvements for parents/caregivers (Postorino et al., 2017 [6]; Rodgers et al., 2021 [7]) are not yet being realized for in-home practitioner-led ABA interventions but do seem possible. The next step would be the thoughtful development of a more hybrid approach that includes parents/caregivers, clarifies expectations, utilizes a collaborative and parent/caregiver supportive approach, and adds validated quantitative measures of outcomes.

## Figures and Tables

**Table 1:** Barriers.

Barrier	Definition
Challenges navigating relationships	Navigating relationships between service providers and caregivers/families which have the potential to be a barrier to service delivery
Cultural barriers	Cultural issues and understanding/misunderstandings which have the potential to disrupt relationships between provider and family
Environmental barriers—home	Distractions in the home
Environmental barriers—school	Service/intervention differences between home and school
Parent/caregiver challenges	Challenges faced when one is the parent/caregiver of an autistic child
Parent/caregiver challenges managing services	Managing the disconnect between home and school and other settings
Lack of parent/caregiver involvement in intervention services	Some parents/caregivers do not get involved in services due to busy schedules or lack of engagement

**Table 2:** Facilitators.

Facilitator	Definition
Building a relationship with family	Building a relationship (building rapport/pairing) between BCBAs, caregivers/families, clients, and RBTs facilitates the delivery of successful services
Coaching parents/caregivers	Instructing parents/caregivers in ABA technique through demonstration, tools, and training facilitates successful services
Communicating with parents/caregivers	Communicating with parents/caregivers, including updates and opportunities for parents/caregivers to ask questions, is a facilitator to successful services. This includes strategies for communicating with each other
In-home services	Benefits of delivering in home settings
Parent/caregiver involvement	Parent/caregiver involvement is a facilitator to successful services

**Table 3:** Recommendations.

Recommendation	Definition
Caregiver training	Recommendations and requests for development of caregiving training, including tools and approaches
Experiences are unique to client/family situation	The process and experience of ABA services are unique to each client and their family, including their needs, what methods and environments are most effective, and how success is measured
Improved communication	Feedback on communication methods and strategies for improvement
Navigator/liaison for families	Navigator or liaison for families to provide them with more information and answers outside of relationship with BCBA/RBTs
Parent/caregiving groups	Requests for parent/caregiver peer groups for support, feedback, and knowledge sharing
Additional programming	Additional ABA services and recreational and other programming
Staff training	More training, mainly focused on navigating relations with parents/caregivers/families

## Data Availability

The datasets presented in this article are not readily available because as a qualitative study with a relatively small sample individual may be identifiable. Requests to access the datasets should be directed to philip.mccallion@temple.edu.
